# Effectiveness of group body psychotherapy for negative symptoms of schizophrenia: multicentre randomised controlled trial^†^

**DOI:** 10.1192/bjp.bp.115.171397

**Published:** 2016-07

**Authors:** S. Priebe, M. Savill, T. Wykes, R. P. Bentall, U. Reininghaus, C. Lauber, S. Bremner, S. Eldridge, F. Röhricht

**Affiliations:** **S. Priebe**, FRCPsych, **M. Savill**, PhD, Unit for Social and Community Psychiatry, WHO Collaborative Centre for Mental Health Services Development, Queen Mary University of London, London, UK; **T. Wykes**, PhD, Institute of Psychiatry, Kings College London, London, UK; **R. P. Bentall**, PhD, Department of Psychiatry, University of Liverpool, Liverpool, UK; **U. Reininghaus**, MSc, DiplPsych, Institute of Psychiatry, Kings College London, London, UK and Department of Psychiatry and Psychology, School for Mental Health and Neuroscience, Maastricht University, The Netherlands; **C. Lauber**, MD, Services Psychiatriques, Jura Bernois – Bienne-Seeland, Bellelay, Switzerland; **S. Bremner**, PhD, Brighton and Sussex Medical School, University of Sussex, Brighton, UK; **S. Eldridge**, MSc, Centre for Primary Care and Public Health, Queen Mary University of London, London, UK; **F. Röhricht**, MD, MRCPsych, Unit for Social and Community Psychiatry, WHO Collaborative Centre for Mental Health Services Development, Queen Mary University of London, London, UK

## Abstract

**Background**

Negative symptoms of schizophrenia have a severe impact on functional outcomes and treatment options are limited. Arts therapies are currently recommended but more evidence is required.

**Aims**

To assess body psychotherapy as a treatment for negative symptoms compared with an active control (trial registration: ISRCTN84216587).

**Method**

Schizophrenia out-patients were randomised into a 20-session body psychotherapy or Pilates group. The primary outcome was negative symptoms at end of treatment. Secondary outcomes included psychopathology, functional, social and treatment satisfaction outcomes at treatment end and 6-months later.

**Results**

In total, 275 participants were randomised. The adjusted difference in negative symptoms was 0.03 (95% CI −1.11 to 1.17), indicating no benefit from body psychotherapy. Small improvements in expressive deficits and movement disorder symptoms were detected in favour of body psychotherapy. No other outcomes were significantly different.

**Conclusions**

Body psychotherapy does not have a clinically relevant beneficial effect in the treatment of patients with negative symptoms of schizophrenia.

Schizophrenia is a severe mental health disorder that affects approximately 0.7% of the population.^1^ Symptoms include positive symptoms such as hallucinations, disordered thinking and delusions, and negative symptoms that include expressive deficits such as blunted affect and impoverished speech, and experiential deficits such as asociality, anhedonia, and avolition.^2,3^ Negative symptoms have been found to have a profound impact on long-term outcomes,^4,5^ but current treatment options are limited. In a review by the National Institute for Health and Care Excellence (NICE) in the UK,^6^ arts therapies – an umbrella term for all non-verbal creative therapies such as art therapy, music therapy and body psychotherapy – were identified as the only type of therapy with justified claims to reduce negative symptoms. Consequently, it was recommended that clinicians should consider referring people with schizophrenia for arts therapies.^6,7^ However, the review was based on only six small-scale trials, meaning more evidence is needed. Since the publication of NICE guidelines one large trial of conventional art therapy has been completed (MATISSE) that found no significant treatment effect on negative symptoms.^8^ Following MATISSE, the aim of the present study was to evaluate the effectiveness of a different type of arts therapy, namely body psychotherapy, as a treatment for negative symptoms of schizophrenia. Body psychotherapy is a form of therapy that involves an explicit theory of body–mind functioning designed to improve emotional, cognitive, physical and social integration. In an earlier trial where this therapy was evaluated,^9^ a significant reduction in negative symptoms was detected in the body psychotherapy group in comparison with a supportive counselling control group. The effect size was large, and was maintained months later. However, this study was relatively small (45 participants), did not control for the non-specific effects of supported group physical activity, and all body psychotherapy groups were conducted by the same therapist. Three earlier trials on body-oriented psychotherapy not included in the NICE review suggested improvements in various outcomes including negative symptoms,^10–12^ however, all had significant methodological shortcomings.

There are a number of advantages to evaluating this particular form of arts therapy as a treatment for schizophrenia. First, it is recognised that patients with schizophrenia can experience a range of body disturbances such as desomatisation, abnormal bodily sensations and motor impairments.^13,14^ Consequently, providing a form of therapy that focuses on the body may help to address such disturbances. Second, to our knowledge this is the only form of arts therapy where a treatment manual specific to the treatment of negative symptoms has been produced that details a theoretical model, mode of action and a standardised therapy structure. Beyond its possible clinical effectiveness, body psychotherapy is relatively inexpensive, can be combined flexibly with other treatment methods, and may appeal to patients who are difficult to engage in other treatments given its novel approach. In order to examine the effectiveness of body psychotherapy as a treatment for negative symptoms we conducted a full-scale, randomised controlled trial (RCT) comparing a manualised form of the intervention with a well-defined, physically active control condition, namely Pilates. Pilates is a structured physical fitness programme involving stretching and controlled movement. The specific components of body psychotherapy under investigation were the focus on body experience at a cognitive and emotional level, the facilitation of emotional group interactions, and the link between movement and emotion. The components common to both interventions include the non-specific effects on non-emotional group interactions, group facilitator attention and physical activity.

## Method

### Design and participants

This study was an assessor-masked, two-arm, RCT, approved by the Camden and Islington National Research Ethics Committee (Ref:H0722/44), and the trial is registered (ISRCTN84216587). A detailed study design description is available in the published protocol.^15^ Participants were recruited from mental health community services in the UK. The inclusion criteria were: diagnosis of schizophrenia (ICD-10 codes F20.0–F20.9),^16^ aged 18–65 years, a Positive and Negative Syndrome Scale (PANSS) negative subscale score ➮18,^17^ no change of antipsychotic medication for 6 weeks, a willingness and ability to consent and participate, and a sufficient command of English to complete the research interviews and actively participate in group interactions in English. Participants were randomised into a manualised, 20-session body psychotherapy group, or a 20-session beginner's-level Pilates class, in addition to standard care.

### Randomisation and masking

Randomisation was conducted by the Pragmatic Clinical Trials Unit (PCTU) independently through a computer-generated sequence. Participants were randomly allocated, with equal probability, to the intervention or control group, stratified by study centre, in batches using randomly permuted blocks of four and six, starting each batch at the start of a new block to preserve balance. The chief investigator, all assessors and the trial statistician were masked to the treatment allocation until all end-of-treatment data were collected and the statistical analysis plan was signed off. To maintain masking, baseline assessments took place prior to randomisation.

### Outcomes

Outcomes were assessed at baseline, end of treatment and 6 months after treatment completion. The primary outcome was the PANSS negative subscale^17^ at the end of treatment. Secondary outcomes were general psychopathology and positive symptoms measured with the PANSS;^17^ the Clinical Assessment Interview for Negative Symptoms (CAINS)^3^ expression and experience subscales; subjective quality of life using the Manchester Short Assessment of Quality of Life (MANSA);^18^ objective social situation using the SIX;^19^ depression using the Calgary scale;^20^ the number of social contacts using the Social Network Scale (SNS);^21^ and a measure of patient activity using four items from the Time Use Survey (TUS).^22^ All were completed at each assessment point. Treatment satisfaction was measured at end of treatment using the Client Satisfaction Questionnaire (CSQ).^23^ Extrapyramidal symptoms (EPS) were evaluated at each stage using the Simpson Angus Scale (SAS),^24^ however, given logistical constraints three items were not assessed (leg pendulousness, head dropping and glabella tap). Given evidence that suggests that the PANSS negative subscale includes some items that relate to cognitive, rather than negative symptoms, the alternative PANSS Marder negative symptom subscale was also evaluated.^25^ For an economic evaluation of the intervention (which will be reported elsewhere), data were obtained using the Client Service Receipt Inventory (CSRI)^26^ and the EQ-5D.^27^ Data from the CSRI were used to calculate the defined daily dose (DDD) of prescribed antipsychotic medication. All researchers were trained in conducting the full PANSS assessment prior to assessing patients. The interrater agreement between all researchers conducting PANSS interviews was assessed at the beginning, middle and end of the study to ensure scores remained sufficiently concordant throughout, using videotapes of the assessments.

### Procedure

Potential participants were approached by their clinicians for consent to be contacted by a researcher. If they agreed, the researcher arranged a meeting during which an explanation of the study was provided, informed consent obtained and an eligibility assessment completed. Once approximately 16 eligible participants were recruited a full baseline assessment, which included a second PANSS assessment, was undertaken within 1 month prior to the group start date. The assessments were typically conducted in the participants' home, or the local community mental health team site, and took 40–120 min to complete. Once all baseline assessments were completed a list of identification codes was sent to the PCTU via the trial manager for randomisation, approximately 1 week before the groups were going to start. Participants were then notified of their group allocation by the relevant group facilitators. After group completion participants were assessed at end of treatment, within 1 month of the groups' completion, and again 6 months later. Participants were paid £25 expenses for each assessment attended.

### Experimental and control conditions

The treatment under investigation was body psychotherapy, as outlined in the manual.^9,28^ Body psychotherapy has a long tradition in psychiatry, going back to the beginning of the 20th century, and has been influenced by psychodynamic psychotherapies, dance movement psychotherapy, and techniques designed to address body image disturbances.^29^

The main goals of body psychotherapy as a treatment for negative symptoms in chronic schizophrenia are: to reconstruct a coherent ego structure through grounding and bodily awareness; to strengthen self-referential processes as a prerequisite for safe social interaction and reality testing; to widen and deepen the range of emotional responses to environmental stimuli; to improve boundary demarcation, enabling differentiation between self and other; and to help patients explore a range of expressive and communicative behaviours with the aim of reducing emotional withdrawal and improving prosocial capabilities

Each session comprised five discrete sections. The first section aimed to facilitate communication between patients, and draw patients' focus towards the body. The second section focused on physical experiences and movements, exploring the personal and general space. The third section addressed specific body image disturbances such as boundary loss and desomatisation. The fourth section centred on creativity and tasks requiring patients to use their bodies and movement as a source of expression and pleasure. In the final section, patients reflect on events, thoughts or feelings that may have been brought up by the group.

Both body psychotherapy and Pilates groups were delivered in 20 sessions of 90 min each, over a 10-week period, held twice a week on non-consecutive days. This duration of treatment was deemed appropriate given the therapy had been manualised for 20 sessions, was long enough to result in significant medium and large treatment effects in two recent trials of body psychotherapy,^9,30^ and in a review on music therapy 16 sessions were sufficient to result in medium-effect size improvements in negative symptoms.^31^ Groups contained between seven and ten participants. To limit the impact of any one therapist or instructor each one ran a maximum of two groups.

Each body psychotherapy group was facilitated by an Association of Dance Movement Psychotherapy (ADMP) accredited therapist trained to deliver the manualised intervention, supported by a co-facilitator. Each therapist received three supervision sessions held by a senior therapist per group. Adherence to the manual was assessed using an adherence scale that we produced for this study (see online supplement DS1) by therapists evaluating four randomly selected sessions of each group (one from each quartile). The scale considered both the content of the sessions, assessing whether therapists adhered to the format and utilised the techniques and objects appropriately, and their competence, assessing their ability to foster a cohesive therapeutic environment and their ability to translate the activities undertaken as strategies to address specific negative symptoms.

The active control condition was beginner's Pilates classes, which was described to participants as a physical health and fitness intervention. All classes were facilitated by a Register of Exercise Professionals (REPS) level-three qualified Pilates instructor, assisted by a co-facilitator. Prior to starting, instructors received a brief training session from an experienced clinician. A brief Pilates guide was developed based on the Pilates Union Matwork Manual.^32^ The guide provided a summary of how to run the groups, and a loosely structured exercise plan. Props (other than mats and head blocks), music and activities designed to encourage group interactions was not permitted. Group interactions were expected to occur as they commonly do when people conduct activities in a group. However, instructors were advised not to initiate or promote any interactions.

### Analysis plan

A 20% reduction in the PANSS score has been used as an indicator of clinically significant improvement in the past, which would be a difference of approximately three points given the eligibility criteria. To detect this difference with a standard deviation of 5, with 90% power for 5% significance, 58 patients were required in each arm. To allow for clustering by group, an intracluster correlation coefficient (ICC) for treatment group of 0.1, and seven patients per group with analysable data at the end of treatment gives an inflation factor of 1.6, meaning 93 participants in each arm were required. At 6 months we anticipated a 31% drop-out, so recruiting 256 participants would leave 88 per arm at 6 months, and 91% power to detect a difference of three points. A total of 128 patients per arm, i.e. 16 groups of approximately 8 patients in each arm, gave 94% power for the end-of-treatment analysis, assuming 87.5% of patients have analysable data. Estimates for the standard deviation ICC for treatment and study drop-out were based upon the findings from the exploratory trial.^9^

The primary outcome was the PANSS negative subscale at the end of treatment, using an available case analysis following intention-to-treat principles. Mixed-effects models fitted by restricted maximum likelihood with fixed effects for the intervention, baseline PANSS negative scores and centre, and random effects for therapy groups were used. To evaluate the impact of missing data, multiple imputation of the data-set was performed and the analysis replicated. A simple complier-average causal effect (CACE) analysis was completed,^33^ defining adherence as attending at least five body psychotherapy sessions. Planned subgroup analyses examined whether there were differences in response between those with higher negative symptoms at baseline, and a longer duration of illness. Analyses were completed using Stata version 12.

## Results

**Fig. 1:**
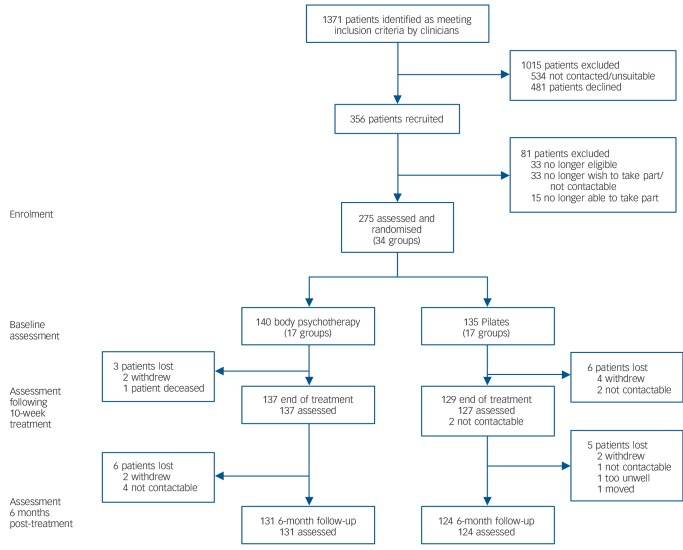
CONSORT diagram.

In total 275 participants were randomised, recruited from December 2011 until June 2013. The study attrition rate for both groups was low; however, the rate of screening required to identify eligible participants was higher than anticipated (see Fig. 1). This was because of a number of factors. First, a relatively large number of screened patients were found to be ineligible, because of either an incorrect diagnosis or insufficient negative symptoms. Second, once all group therapy/control places were provisionally filled, no more participants were approached unless a participant subsequently dropped out. Consequently, a number of participants were initially screened as potentially eligible, but were not approached as a result of the lack of available spaces on the trial in their area. Of those randomised, 266 (96.7%) were assessed at end of treatment, and 255 (92.7%) went on to complete the 6-month follow-up.

The baseline characteristics of the sample are presented in Table 1. Participants presented with moderate levels of negative symptoms (PANSS negative score 23.1, s.d. = 4.4). The mean level of interrater reliability for the PANSS was high (PANSS total intraclass coefficient 0.85). Assessor masking was maintained prior to the primary outcome assessment in 94.3% of cases.

**Table 1:** Descriptive statistics of participant characteristics at baseline, for experimental and control condition

Variable	Body psychotherapy group (*n* = 140)	Pilates group (*n* = 135)	Total (*n* = 275)
Centre, *n* (%)			
East London^a^	41 (29)	40 (30)	81 (29)
North East London^a^	8 (6)	8 (6)	16 (6)
South London	36 (26)	32 (24)	68 (25)
Manchester	23 (16)	23 (17)	46 (17)
Liverpool	32 (23)	32 (24)	64 (23)

Age, years: mean (s.d.)	41.1 (10.1)	43.3 (11.1)	42.2 (10.7)

Gender, *n* (%)			
Men	103 (74)	100 (74)	203 (74)
Woman	37 (26)	35 (26)	72 (26)

Ethnicity, *n* (%)^b^			
White	71 (52)	67 (53)	138 (52)
Black	39 (29)	38 (30)	77 (29)
Asian	13 (9)	16 (13)	29 (11)
Other	14 (10)	6 (5)	20 (8)

Employment, *n* (%)^c^			
Unemployed	131 (94)	132 (98)	263 (96)
Other	8 (6)	3 (2)	11 (4)

Living situation, *n* (%)^c^			
Alone	83 (60)	73 (54)	156 (57)
With others	56 (40)	62 (46)	118 (43)

Number of children, median (IQR)	0 (0–1)	0 (0–1)	0 (0–1)

Duration of illness, median (IQR)	12.6 (8.8)	12.7 (9.5)	12.6 (9.1)

Number of hospital admissions, median (IQR)	3.9 (3.8)	4.0 (4.2)	4.0 (4.0)

Medication: defined daily dose, mean (s.d.)	1.48 (1.11)	1.71 (1.28)	1.59 (1.20)

IQR, interquartile range.

a. These two centres were treated as one for the purposes of the stratified randomisation.

b. As a result of missing data total *n* for the body psychotherapy group is 137, for the Pilates group 127 and for all participants 264.

c. As a result of missing data total *n* for the body psychotherapy group is 139 and for all participants 274.

Participants attended significantly more body psychotherapy sessions (body psychotherapy median 11, interquartile range (IQR) = 5–17; Pilates median 8, IQR = 1–15; *P* = 0.01). In total, 106 participants (75.7%) attended at least five body psychotherapy sessions, the level defined as adhering to treatment in the CACE analysis. Therapist adherence to the manual was relatively high with a mean score of 17.6 (out of 20; s.d. = 0.21).

### Primary outcome

Outcomes are shown in Table 2. There was a small reduction in mean PANSS negative symptoms between baseline and end of treatment in both groups (within-group mean reduction in the body psychotherapy group 1.5 (s.d. = 3.5); Pilates group 1.6 (s.d. = 3.8)). After controlling for baseline scores, study centre and therapy group, no significant difference between the experimental and control condition was detected (adjusted mean difference = 0.03, 95% CI −1.11 to 1.17, *P* = 0.959. Model-based ICC = 0.099).

**Table 2:** Descriptive statistics and complete case analysis of outcome measures over the three time points by condition

	Body psychotherapy group			Pilates group			Between-group differences^b^				
Outcome^a^	Baseline	End of 10-week treatment	6 months post- treatment	Baseline	End of 10-week treatment	6 months post- treatment	At end of treatment		6 months post-treatment	
							Adjusted mean differ- ence/lRR (95% CI)^c^	ICC^d^	Adjusted mean differ- ence/lRR (95% CI)^c^	ICC^d^
PANSS, mean (s.d.)										
Negative (*n* = 254)	23.3 (4.3)	21.8 (5.4)	21.7 (5.7)	23.1 (4.4)	21.5 (4.7)	21.7 (5.1)	0.03 (−1.11 to 1.17)	0.099	−0.18 (−1.68 to 1.31)	0.137
Positive (*n* = 253)	14 (5.1)	13.1 (4.7)	13.4 (4.7)	14.1 (4.7)	13.3 (4.2)	13.6 (4.9)	0.06 (−0.71 to 0.84)	<0.001	−0.12 (−1.03 to 0.79)	<0.001
General (*n* = 249)	32.9 (8.3)	30.2 (8)	30.1 (8.1)	32.5 (8.1)	29.9 (7.3)	30.4 (7.5)	0.32 (−1.31 to 1.94)	0.096	−0.70 (−3.07 to 1.67)	0.205
Marder negative (*n* = 253)	22.2 (4.7)	20.7 (5.7)	20.2 (5.7)	21.9 (5)	20.3 (5.1)	20.1 (5.6)	0.23 (−0.86 to 1.32)	0.678	0.04 (−1.38 to 1.45)	0.075

CAINS mean (s.d.)										
Experience (*n* = 246)	22.1 (5.6)	20.5 (5.8)	20.8 (6.7)	21.5 (5.5)	19.8 (5.8)	20.6 (6.2)	0.05 (−1.13 to 1.22)	0.037	−0.04 (−1.48 to 1.40)	0.041
Expression (*n* = 253)	8 (3.5)	7.3 (3.7)	7.1 (4)	7.5 (3.9)	7.5 (4.1)	7.1 (4.3)	−0.62 (−1.23 to 0.00)	0.022	−0.27 (−1.05 to 0.50)	0.023

Calgary, mean (s.d.) (*n* = 253)	4.8 (4.2)	3.9 (4.3)	4.1 (4.1)	4.6 (4.6)	3.9 (4.3)	4.2 (4.2)	−0.01 (−0.72 to 0.71)	<0.001	−0.20 (−1.18 to 0.79)	0.086

MANSA, mean (s.d.) (*n* = 254)	4.4 (0.9)	4.5 (0.9)	4.6 (1)	4.4 (0.9)	4.6 (0.9)	4.5 (0.9)	−0.11 (−0.27 to 0.58)	<0.001	0.10 (−0.12 to 0.32)	0.050

SNS, median (IQR) (*n* = 232)										
Relatives seen	2 (1.0–3.0)	3 (1.0–4.0)	2 (1.0–4.0)	2 (1.0–4.0)	2 (1.0–4.0)	2 (1.0–4.0)	1.13 (0.89 to 1.32)	–	0.96 (0.80 to 1.15)	–
Friends seen	1 (0.0–2.0)	1 (0.0–2.0)	0.5 (0.0–2.0)	1 (0.0–2.0)	1 (0.0–2.0)	1 (0.0–2.0)	0.94 (0.80 to 1.42)	–	0.91 (0.63 to 1.30)	–
Total number seen	3 (2.0–5.0)	4 (3.0–6.0)	4 (2.0–6.0)	4 (2.0–5.0)	4 (3.0–6.0)	4 (2.0–6.0)	0.83 (0.69 to 1.14)	–	0.97 (0.85 to 1.12)	–

TUS, median (IQR) (*n* = 254)										
Number of activities	3 (1.0–6.0)	3 (1.0–7.0)	3 (1.0–7.0)	3 (1.0–6.0)	2 (1.0–7.0)	2 (1.0–7.0)	1.03 (0.89 to 1.42)	–	1.04 (0.81 to 1.33)	–
Time spent (hours)	1.5 (0.0–3.5)	1.5 (0.3–4.0)	1.5 (0.0–3.0)	1.8 (0.3–4.0)	2 (0.3–4.5)	1.5 (0.2–3.8)	1.03 (0.80 to 1.32)	–	0.96 (0.73 to 1.25)	–

SAS, mean (s.d.) (*n* = 229)	1.7 (2.1)	1.2 (1.7)	1.2 (1.5)	2.3 (2.7)	2.1 (2.9)	1.9 (2.4)	−0.65 (−1.13 to −0.16)	<0.001	−0.50 (−0.94 to −0.07)	0.007

SIX, mean (s.d.) (*n* = 254)	2.4 (1.1)	2.5 (1.1)	2.5 (1)	2.3 (1.1)	2.5 (1.1)	2.5 (1.2)	−0.02 (−0.17 to 0.20)	<0.001	−0.10 (−0.27 to 0.08)	<0.001

CSQ, mean (s.d.) (*n* = 237)	–	25.3 (4.6)	–	–	25.9 (4)	–	−0.68 (−1.80 to 0.44)	–	–	–

PANSS, Positive and Negative Syndrome Scale; CAINS, Clinical Assessment Interview for Negative Symptoms; Calgary, Calgary Depression Scale; MANSA, Manchester Short Assessment of Quality of Life; SNS, Social Network Scale; TUS, Time Use Survey; SAS, Simpson Angus Scale; CSQ, Client Satisfaction Questionnaire.

a. The number of participants completing each outcome at 6 months (and for the CSQ at end of treatment) is included in brackets.

b. Models adjusted for baseline measure of outcome, study centre and a random effect for therapy group (except CSQ).

c. All values are adjusted mean difference except those for SNS and TUS where incident rate ratios (IRRs) are reported.

d. Values are model-based intracluster correlation coefficients (ICCs). ICCs not calculated for count outcomes.

### Secondary outcomes

A significant mean difference reduction in the SAS (−0.65, 95% CI −1.13 to −0.16, *P* = 0.009, ICC<0.001), which measures extrapyramidal symptoms, and the CAINS expression subscale (−0.62, 95% CI −1.23 to 0.00, *P* = 0.049, ICC = 0.022), which measures asociality, anhedonia and avolition was detected in the body psychotherapy arm in comparison with the Pilates group at the end of treatment. No other significant differences were found at this stage. In an analysis of the multiply imputed datasets, no substantial differences in the results were evident, although the reduction in the CAINS expressive subscale was no longer below the *P* = 0.05 threshold for significance (−0.60, 95% CI −1.22 to 0.02, *P* = 0.056, ICC = 0.026).

At the 6-month follow up, no significant mean difference in the PANSS negative score was detected between conditions (−0.18, 95% CI −1.68 to 1.31, *P* = 0.812, ICC = 0.137). There was a significant mean difference in the SAS at 6-month follow up (−0.50, 95% CI −0.94 to −0.07, *P* = 0.028, ICC = 0.007), but no other significant differences were detected. In the CACE analysis, no significant mean difference in the PANSS negative score between body psychotherapy and Pilates was detected (−0.13, 95% CI −1.41 to 1.64). In the secondary outcomes, only a significant mean difference in the SAS was detected (−0.82, 95% CI −1.51 to −0.12). As an exploratory outcome, an additional CACE analysis on the primary outcome was conducted where those adherent to treatment were defined as those that attended at least 10 body psychotherapy sessions, and again no significant difference was detected (0.15, 95% CI −1.89 to 2.19). In pre-planned subgroup analyses, no significant differences in response were detected between patients with higher negative symptoms at baseline, or a longer duration of illness. No serious adverse events related to either intervention were reported.

## Discussion

No significant differences between body psychotherapy and Pilates were detected in the PANSS negative symptom subscale. A statistically significant improvement in the body psychotherapy arm was detected in the CAINS expression subscale and movement disorder symptoms. However, the small effect sizes mean these improvements are unlikely to reflect relevant clinical benefits. There was no significant difference on other outcomes. Given that the confidence interval excludes a clinically meaningful difference in negative symptoms on the PANSS, and the high statistical power, these results support the conclusion that body psychotherapy is not an effective treatment for patients with negative symptoms of schizophrenia as compared with Pilates as an active control.

### Strengths and limitations

The study retention rates were excellent, with 92.7% of participants remaining in the study until its end. The large sample sizes and minimal drop-out meant the study was highly powered to detect a clinically important difference in the primary outcome (>94%). This, together with the non-significant result suggest that these findings go further than just failing to reject the null hypothesis, and instead can be interpreted as evidence of the intervention having no clinically important benefit.

The intraclass coefficient scores on the PANSS between assessors was high (PANSS intraclass coefficient 0.85), with no evidence of rater drift. Participants randomised to body psychotherapy attended a median of 11 sessions, which is relatively high given participants typically experienced high social withdrawal and motivation deficits. Approximately 40% of participants in the body psychotherapy condition attended at least 75% of the sessions offered, which compares very favourably with the MATISSE trial evaluating art therapy with a similar patient group.^8^ The Pilates groups were also well attended, enabling a comparison that appropriately controls for the non-specific effect of regular group activity. This relatively high attendance is likely to be attributable to the logistical support provided by the co-facilitators, which included the provision of taxis to those who required it. The body psychotherapy intervention was manualised and therapists were largely adherent to treatment guidelines, allowing the intervention to be evaluated as it had been designed.

One limitation is that in the Pilates groups emotional group interactions, although discouraged, may also have occurred. In addition, although the focus on body experience at a cognitive and emotional level may not be explicitly addressed in Pilates, an emphasis on centring, concentration and breathing may have implicitly fostered such links. A link between movement-based exercises such as Pilates and mindfulness, which may help address negative symptoms, has been proposed.^34,35^ However, the small within-group changes detected suggest that neither group was effective, as opposed to both being equally effective. A pre–post reduction of 1.5 points in the PANSS negative subscale was half the level prespecified as an indicator of clinically meaningful change, and was comparable with the 1.3 point reduction found in the supportive counselling group evaluated in the exploratory trial.^9^ The reduction found is consistent with changes in treatment-as-usual study arms in a recent meta-analysis that examined the within-group changes of negative symptoms over time,^36^ suggesting the improvements observed were spontaneous, and did not reflect any therapeutic effect. Given the symptom change in the Pilates group was similar to control conditions from other clinical trials that aimed to treat negative symptoms, it suggests that adopting Pilates as a comparator was appropriate, with the findings generalisable to other active control conditions presuming they do not provide any additional clinical benefit over treatment as usual either.

Another possible limitation is the relatively short duration of the treatment under investigation. Although it remains unclear whether more prolonged exposure to therapy may result in changes to negative symptoms, this should be considered unlikely. In the meta-analysis on the dose–response effect of music therapy,^31^ the relationship between the number of sessions and improvements in negative symptoms was curvilinear, with small effect sizes found in as few as three sessions, and medium effects in 16 sessions. In the prespecified CACE analysis no significant differences between the groups were detected when those adherent to treatment were defined as attending at least five sessions. In an exploratory analysis of our data the difference was also highly non-significant when this threshold was increased to a minimum attendance of ten sessions. If the lack of effect is attributable to insufficient dose, it would be reasonable to expect at least a trend towards symptom improvements as participants received more sessions, however, this was not detected.

### Comparisons with existing literature

These findings are in contrast to the exploratory trial where significant improvements in negative symptoms were found in the body psychotherapy group compared with supportive counselling.^12^ This study was the only one identified of sufficient quality to be included in a recent Cochrane review of dance therapy for schizophrenia.^37^ In the context of arts therapies as a whole, our findings contradict the current NICE arts therapy review,^6^ instead mirroring those reported in the MATISSE art therapy trial.^8^ Collectively, these two trials could be considered to have one of two implications for the NICE recommendations, dependent upon how the concept of arts therapies itself continues to be defined. If we continue to evaluate arts therapies as a singular treatment ‘type’ as is the case in the present NICE review,^6,7^ then incorporating the findings from the current study and MATISSE would result in the current evidence base suggesting that arts therapies are not an effective treatment for negative symptoms of schizophrenia. If arts therapies are instead recognised as heterogeneous, each with a different model of action, then it suggests that the current evidence base upon which NICE concludes that arts therapies may be helpful for negative symptoms may be inappropriate. If we presume the latter, then full-scale trials in other arts therapies such as music therapy may be merited in this particular patient group. Although small-scale investigations have suggested that music therapy may be effective,^38^ given that promising results in small-scale investigations have not been replicated either here or in MATISSE, it suggests that caution should be advised in interpreting such findings.

In the secondary outcomes a small, significant improvement in the body psychotherapy group was detected in expressive symptoms at end of treatment measured by the CAINS, and in movement disorder symptoms both at end of treatment and 6 months later. For both findings it is important to consider that multiple testing with the risk of an inflated type I error was conducted. However, the fact that a difference was detected in this scale, in contrast with the PANSS, may be important given one of the main aims of the Collaboration to Advance Negative Symptom Assessment for Schizophrenia (CANSAS)^39^ was to develop new scales that are sufficiently sensitive to detect negative symptom change in clinical trials.^2^ Second, this finding may provide further evidence for the importance of measuring expressive and experiential features of negative symptoms separately given they represent separate constructs.^40^ The change in movement disorder symptoms should be interpreted with much caution since an incomplete scale was used. Although it is intuitive to consider that a treatment that focuses specifically on the body may help alleviate movement-related symptoms, this finding should be re-examined in a trial focused on such outcomes before drawing firm conclusions.

In conclusion, overall, this study does not support group body psychotherapy as a treatment for negative symptoms of schizophrenia. Reviewing the effectiveness of different arts therapy modalities separately may be informative to determine whether existing guidelines should be more cautious in recommending art and body psychotherapy specifically, or whether this extends to arts therapies as a whole.
